# Cytochrome *b* and Molecular Typing of *Leishmania* spp. in a Passive Sampling of Suspected Patients with Cutaneous Leishmaniasis in Sistan and Baluchestan Province, Eastern Iran

**Published:** 2017

**Authors:** Gholamreza MOTALLEB, Hadi MIRAHMADI, Ahmad ZARE-ZADEH, Ahmad MEHRAVARAN

**Affiliations:** 1. Dept. of Biology, Faculty of Sciences, University of Zabol, Zabol, Iran; 2. Infectious Diseases and Tropical Medicine Research Center, Resistant Tuberculosis Institute, Zahedan University of Medical Sciences, Zahedan, Iran; 3. Dept. of Parasitology and Mycology, Faculty of Medicine, Zahedan University of Medical Sciences, Zahedan, Iran; 4. Dept. of Biology, Faculty of Sciences, University Campus, Graduate School, University of Zabol, Zabol, Iran

**Keywords:** *Leishmania major*, *Leishmania tropica*, Cytochrome *b*, PCR, DNA sequencing, Iran

## Abstract

**Background:**

Despite the high prevalence and drug resistance of disease in Sistan and Baluchestan Province of Iran, the species of cutaneous leishmaniasis (CL) has not been identified. In the present study, cytochrome *b* (Cyt *b*) was used in Sistan and Baluchestan to find species of *Leishmania* in suspected patients of CL using PCR-RFLP and DNA sequencing.

**Methods:**

This study was conducted from Oct 2015 to Oct 2016. The samples were collected from the individuals clinically suspected to CL and referred to Iran Shahr, Chabahar, Khash, Zabol, Zahedan, Mirjaveh, and Nikshahr health centers. Overall, 700 Giemsa-Stained slides from the wound of patients suspected of CL were passive collected and examined under a light microscope at ×1000. After DNA extraction, positive samples were used for Cyt *b* detection by PCR-RFLP to determine the parasite species. One hundred positive slides were selected for molecular studies. Among positive samples, 20% were sequenced. To compare the results of sequences, molecular evolutionary genetic analysis (MEGA6) was used.

**Results:**

Overall, 53 samples were identified as *L. major* and 47 samples (47%) *L. tropica*. Cyt *b* in *L. major* and *L. tropica* is converted to 400 and 480 bp and 130, 215 and 535 bp pieces respectively. In the isolated *L. tropica* and *L. major*, nucleotide changes were 3–5 (mainly in wobble site).

**Conclusion:**

Infection was more related to *L. major*. PCR-RFLP method has a high sensitivity for diagnosis of *Leishmania* species.

## Introduction

Leishmaniasis is a common zoonotic disease and has an annual incidence of 900000-1300000 new cases and 20000-30000 mortality rate (1). Leishmaniasis is a zoonotic disease that occurs in three types: Cutaneous leishmaniasis (CL), Visceral (Kalazar), and Mucocutaneous. CL is a protozoan from Flagellate group, of Trypanosomatid family and *Leishmania* genus that transfers to human by mosquito bite of Psychodidae family, a subfamily of Phlebotomine of the animal reservoir (mainly rodents and domestic and wild carnivores) and human reservoir.

Its symptoms include wounds that remain in the body for one year (2, 3). This disease is one of the most important and the most common endemic disease in Iran and the second contagious parasitic disease transferred by arthropods after malaria, observed in two rural and urban types. Annually about 20000 cases of CL are reported from different parts Iran and the real value is estimated greater than its reported value (4).

Cytochrome *b* (Cyt *b*) of the mitochondrial genome is very useful for phylogenetic study (5). This gene is located at cytoplasmic helix with 50 copies. Cyt *b* enables identification of 13 common species of Leishmaniasis etiologic factor in human and has the required diversity in nucleotide sequence among genomes of *Leishmania* species for identification. On the other hand, the polymorphism observed in DNA sequence of Cyt *b* prevents the selection of a common probe to identify all *Leishmania* species in qPCR engineering (5). Cyt *b* is used in vertebrate’s phylogenetic studies, but much less frequently used in invertebrates. This gene has the same level of A/T nucleotide sequence variations and has been used phylogenetically at different levels of the taxonomy (from species to orders).

In the present study, Cyt *b* was used in Sistan and Baluchestan to find species of *Leish-mania* in suspected patients of CL using PCRRFLP and DNA sequencing.

## Materials and Methods

Sistan and Baluchestan province (29° 29′ 32.64″ N, 60° 52′ 0.84″ E) has a population of 2534327 according to the last census located in southeastern Iran with long, hot and dry summers and short winters, however, near the Oman Sea, the weather has a high percentage of humidity.

### Sample Collection

This study was conducted from Oct 2015 to Oct 2016. The samples were collected from the individuals clinically suspected to CL and referred to Iran Shahr, Chabahar, Khash, Zabol, Zahedan, Mirjaveh, and Nikshahr health centers. A questionnaire was completed to record the essential information such as demographic information, sites of the ulcer on the body and history of migration. Overall, 700 samples were collected and carried out for *Leishmania* species isolation. According to the proposed project and statistical analysis of the proposal, a minimum of 69 (20%) positive cases in PCR-RFLP were used to carry out the DNA sequencing to compare the results (however we selected 100 samples).

The study was approved by the Ethical Committee of University of Zabol (Project Code: IR-UOZ94-31) and Zahedan University of Medical Sciences and the Iranian Ministry of Health, Treatment and Medical Training Protection Code of Human Subjects in Medical Research. Written informed consents were obtained from subjects that participated in this study.

### Sample Preparation

Ulcer skin lesions of the patients with suspected CL were cleaned with 96% ethanol. With a sterile lancet from the borders wound (in the center to the periphery), a gap was created under the swelling and some of the serosity was transferred to the slide and ran well by the lancet. Of each ulcer patients, two samples, one for microscopic study and viewing Leishman body and the second one was transferred to the liquid phase NNN (Novy -Mac Neal-Nicolle) and RPMI-1640 medium with 10% inactivated fetal bovine serum (FBS) culture medium to grow and the presence of the parasite DNA extracted bodies.

### Microscopic Examination

Standard light microscopy was directly carried out on the smears. Briefly, the slides were air-dried and fixed with absolute methanol, stained in Giemsa 10% (ARJ Company) and viewed in magnification ×1000 to investigate the presence of the amastigote forms (345 samples were positive).

### DNA Extraction

DNA extraction was carried out on the promastigotes obtained from the culture and slides using DNP^TM^ Kit High yield DNA Purification Kit (CinnaGen Molecular Biology and Diagnostic, Iran) and the High Pure PCR Template Purification kit (Dynabio®, Takapouzist, Iran) according to the manufacturer and was stored at −20 °C.

### PCR-RFLP

In order to identify and detection of the isolated *Leishmania* species, the Cyt *b* region was used. The primers as were described before LCBF1 (5′-GGTGTAGGTTTTAGTTTAGG-3′) and LCBR2 (5′-CTACAATAAACAAATCATAATATACAATT-3′) (6). Reactions were carried out in a final volume of 15 μl containing 2.0 μl of DNA preparation, 7.5 μl Master Mix, 4.5μl-distilled water and 1 μl of each primer. PCR amplifying conditions were: initial denaturation at 94 °C for 5 min, 1 cycles: denaturation at 94 °C for 45 sec, 35 cycles, annealing at 50 °C for 45 sec, 35 cycles, extension at 72 °C for 1 min, 35 cycles and final extension at 72 °C for 5 min, 1 cycles. PCR–RFLP was carried out on PCR products. Briefly, 10 μl of PCR products were digested by 0.5 μl Ssp1 (Thermo Fisher Scientific) enzymes and incubated for 5 h at 37 °C in the manufacturer’s buffer (10 X Reaction Buffer, 5 μl). Restriction fragments were separated on 2% polyacrylamide gel. Reference DNA of *L. major* and *tropica* was used as the positive control. Distilled water, buffer R, and enzyme were used as negative control. The PCR products were visualized using a 2% gel agarose electrophoresis and compared with those from the DNA of *Leishmania* reference strains. In Cyt *b* genes in the *L. major* using Ssp1enzymes two fragments and two sizes 400, 480bp were provided. Besides, *L. tropica* Cyt b gene three sections and sizes 130, 215, 535bp were observed.

### Molecular and statistical analysis

Firstly, the samples were sequenced and analyzed by comparing the results of the gene bank using CLUSTAL W Multiple Sequence Alignment Program, Ver. 1.7 software. The MEGA-6 software was used to compare the sequences. For this purpose, the standard samples of PCR product *L. major* and *L. tropica* were sent for sequencing and after determining the sequence of each sample, sequence of other *Leishmania* species such as *gerbili*, *kiliki*, *arabica*, *etiopica*, and *donovani* related to the Cyt *b* were stored from the Gene Bank in Fasta format and were selected using the CLC DNA Workbench 6.6 online software. Twenty percent of all PCR products were sent to South Korea (Bioneer, Korea) for DNA sequencing. To enter the DNA sequence for all samples, and matching nucleotides with chromatography of each sample, Sequencer Tm 4.4 Software was used. Chi-square test with a confidence interval of 0.95 using SPSS 16 (SPSS Inc. Headquarters the USA) software was carried out to compare the means.

## Results

After sampling and preparation of slides from the wound of the suspected cases in microscopic examination of smear slides stained with Giemsa, 345 of 700 samples were positive. The reason of the lack of observation of amastigotes in rest of the slides can be clinical similarity of appearance of the wound with other skin lesions, microbial and fungal infection, low number of parasites in smears taken from lesions, and weak sampling technique. Since the number of samples from different regions was not the same, so the statistical method was used to collect the appropriate number of the samples, however, the others will be used for future work. Ultimately, 100 positive samples were selected for molecular studies. In this study, targeting Cyt *b* by specific primers techniques and RFLP method, 100 cases were positive.

**Fig. 1:**
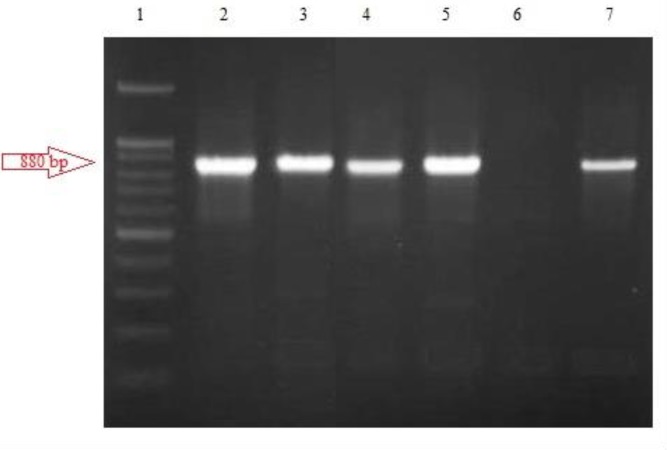
The Cyt *b* electrophoresis Line 1: 100 bp Ladder // Lines 2 to 5: 880 bp bands of suspicious samples // Line 6: negative control //Line 7: positive control

**Fig. 2:**
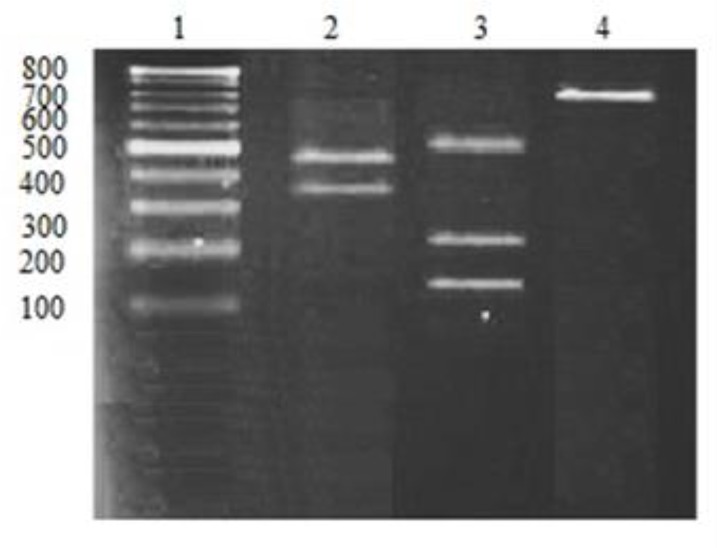
The electrophoresis results of Cyt *b* in *Leishmania* species in the Sistan-Baluchestan province under the effect of *S*sp1 enzymes Line 1: 100 bp Ladder // Line 2: 400 and 480 bp of *L. major //* Line 3: 130, 215 and 535 bp of *L. tropica //*Line 4: 880 bp not under the influence of enzyme.

Higher percent contamination was related to *L. major* (53%) compared with *L. tropica* (47%); these results are consistent with the disease pattern in Sistan and Baluchestan. In this research, PCR products were run on 2% agarose gel and photographed. Proliferated Cyt *b*, weighing 880 bp, in *L. major* cuts into two pieces 400 and 480 bp under the influence of Ssp1 enzyme, and in *L. tropica* into three pieces 130, 215 and 553 bp (Fig. 1 and 2).

**Fig. 3:**
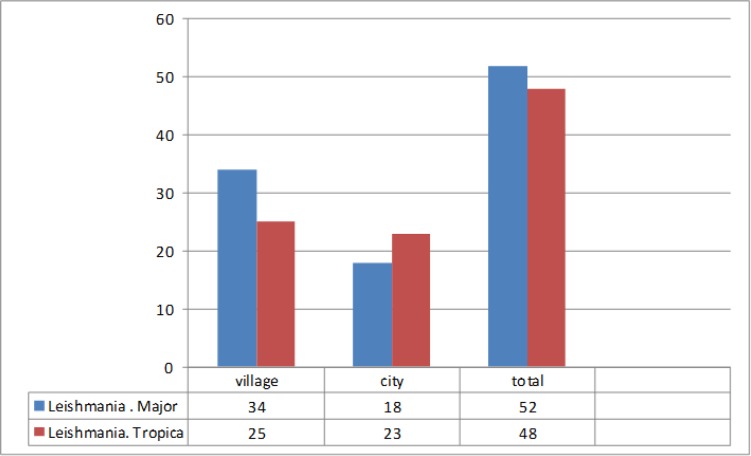
Number of lesions in patients

**Fig. 4:**
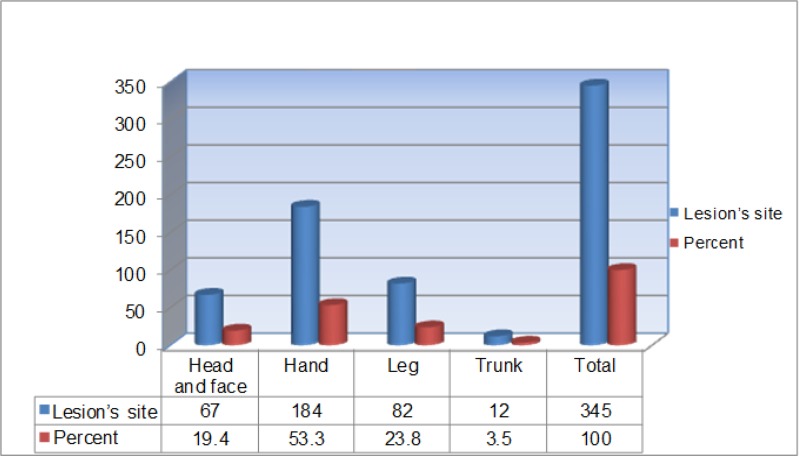
Distribution of wounds based on the lesion’s site

Of the 100 analyzed samples, 66 cases were related to male patients and 34 to females (Table 1). Age of patients with lesions ranged from 1 to over 60 yr. The highest percentage of the suspicious wound in positive direct smear and PCR was related to patients aged 11–20 years. Investigating 100 samples, the maximum number of lesions was related to patients with one wound (51%), and there was no significant relationship between the number of wound and the species of disease agent, based on chi-square test (Fig. 3). Hand and leg had the most frequency of wounds and trunk had the lowest (Fig. 4).

**Table 1:** The results of the species identification based on sex and age groups

***Gender***	***Positive smear cases***		***Positive PCR cases***	
	**Number**	**Percent**	**Percent**	**Number**
Male	246	71.3	66	66
Female	99	28.7	34	34
Total	345	100	100	100
**Age (yr)**	Those with suspicious lesions on positive slides		Positive results in PCR test	
	Number	Percent	Number	Percent
1–10	93	27	25	25
11–20	96	27.8	29	29
21–30	63	18.3	19	19
31–40	51	14.8	17	17
41–50	18	5.2	5	5
51–60	13	3.7	4	4
>60	11	3.2	1	1
Total	345	100	100	100

Among 100 patients with CL, 59 were residents of town and 41 were rural residents and there was no significant correlation between the number of wounds and the disease agent based on chi-square test (Table 2 and 3). For comparing the frequency of *Leishmania* species and residence location (urban/rural), chi-square test was used and the results showed no significant difference between the two groups (*P*=0.29).

**Table 2:** Relative frequency distribution of CL based on the patient's living place

***Location***	***Number***	***Percent of distribution***
Urban	59	59
Rural	41	41
Total	100	100

**Table 3:** Distribution of CL parasite species by geographical region of residence

***Geographical location***	***Number***	***Species***			
		***L. major***	***L. tropica***
Iran Shahr	3	2	1
Chabahar	17	2	15
Khash	7	5	2
Zabol	7	5	2
Zahedan	39	21	18
Mirjaveh	23	21	2
Nikshahr	4	3	1
Total	100	59	41

**Fig. 5:**
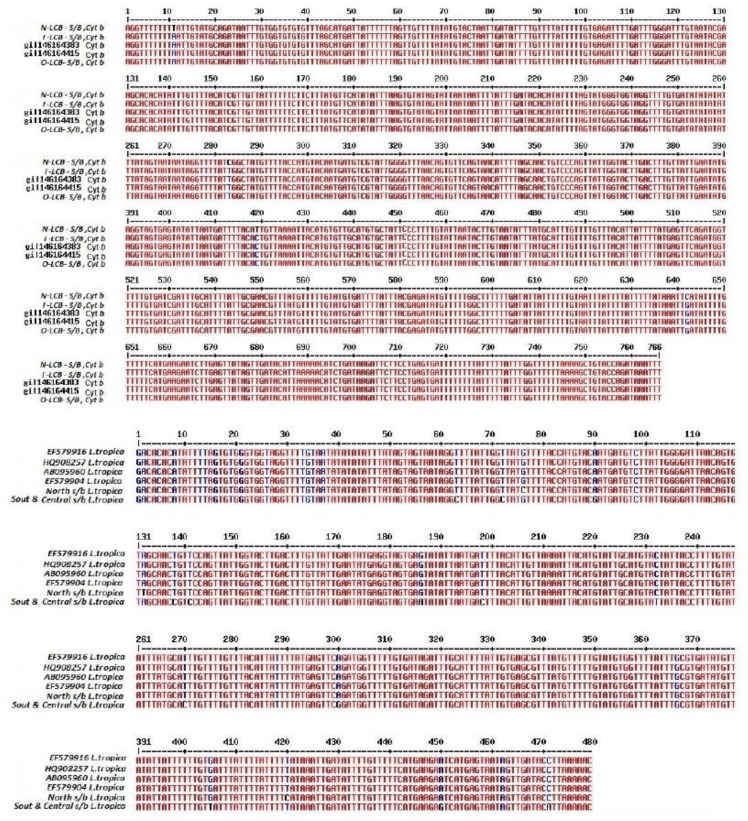
Results of sequencing and sequence analysis

The chi-square test showed that there was significant difference between the two groups acute (rural) and chronic (urban) in both cases of *L. major* and *tropica*. Fig. 5 compares common haplotype sequences of Cyt *b* of *L. tropica* and *major* with haplotypes recorded in the Gene Bank and *L. major* and *L. tropica* isolated from patients in north, south, and center of the province, prepared and compared with the online application. Analysis of sequences determined three nucleotide differences in positions 11, 283, and 419. In the isolated *L. tropica* and *major*, there were 3–5 nucleotide changes between Cyt *b*, predominantly in third place of nucleotide codons (Wobble site) that did not lead to new amino-acid creation. Comparison of sequences of common and new haplotypes of Cyt *b* of *L. major* with haplotypes recorded in Genebank and *L. major* isolated from patients of Zahedan identified 4 different nucleotides in places 11, 283, 419 and 642. Morphometric studies on the shapes and sizes of Leishman bodies in samples taken from the patients showed two round and oval common forms with 2–3 and 3–4 microns. Positive severity-grading results showed that smear of 3+ and 4+ were the most frequent amastigotes. The clinical features of wounds were observed in four categories: combined form (dry and wet), dry, classical wet (volcano view) and non-classical wet (corny, herpes, papol, nodal, erythematous and erysipeloid).

## Discussion

Despite the high prevalence and drug resistance of CL in Sistan and Baluchestan Province, Iran, the species of the parasite has not been identified. At the present study, the PCR-RFLP and DNA sequencing were applied to identify the *Leishmania* species. PCRRFLP method has a high sensitivity for diagnosis of Leishmaniasis and rapid determination of parasite species of the disease.

Cyt *b* has been applied successfully by researchers to classify several species of *Leish-mania* genus. Usually, diagnosis of CL is based on microscopic methods or cultivation of *Leishmania* from skin samples or aspirated wounds. However, these methods are insensitive and time-consuming (7); for example several *Leishmania* species may be simultaneously present in one area (8).

In this case, comparison of ISO enzymes and using DNA of the parasite is the gold standard to differentiate the species of *Leish-mania* (9). Determination of *Leishmania* species based on clinical signs and symptoms can be problematic; for example, CL needs differential diagnosis due to diverse clinical features (10). The common PCR method for detection and identification of *Leishmania* is more sensitive than conventional direct microscopic observation and in vitro culture, although it increases the risk of false-positive results due to an increased risk of sample contamination (Liquid handling). Several types of DNA targets have been used to identify the type of Leishmania using various methods such as PCR, including rRNA genes, kinetoplast DNA, and 18S rRNA gene sequences. All of these genes are recurring in a variety of *Leishmania* and therefore increase the detection sensitivity.

However, the species have been determined based on sequencing Cyt *b*. This issue is importantly practical for passengers and people with underlying CL because the extracted *Leishmania* in these patients usually belong to different species. For example, unlike Africa, where *L. major*, *L. tropica* and *L. infantum* are rarely endemic in one area, different types of CL overlap in South America and Middle East. A passenger who may return from a particular region of Peru may be affected with the following species: *L. braziliensis, L. panamensis, L. guyanensis, or L. amazonensis. L. braziliensis,* while *L. panamensis* and *L. guyanensis* is often limited with few number of injections or even with one pentamidine injection or oral administration of miltefosine. Therefore, it is necessary to determine the species. In order to determine the type and species of *Leishmania* parasite, several techniques and laboratory methods, such as RFLP after the PCR, have been reported, but this technique does not produce results to be used in different laboratories and changing data to be used in a computer.

On the other hand, the main reason for the lack of selection of Cyt *b* is its position in areas with the largest ring mode in kinetoplast, where there are about 50 copies of it. On the other hand, the required nucleotide diversity in the genome of *Leishmania* species, such as polymorphic nucleotide in 245 position and 190 positions of parsimony is informative and is thus successfully used in Argentina and Pakistan to determine the species of *Leishmania*. In the present study, we successfully used Cyt *b* genes using PCR-RFLP and DNA sequencing on 100 cases of clinically diagnosed CL to recognize parasite species. As this method can be used both for positive and microscopic samples to determine the species. *L*. *major* causes wet CL in Iran and dry CL is caused by *L. tropica* (11).

The serological methods have less ability for diagnosis of cutaneous Leishmaniasis of urban and rural type. Diagnosis of *Leishmania* is, however, easily possible by providing slides from the patient’s wound and Giemsa staining, but diagnosis will be difficult in the case of chronic wound, where the parasite numbers are low (12). Kinoplastic DNA-based PCR methods for *Leishmania* are sensitive tests for the diagnosis of disease, especially in chronic cases (13). Today, to develop a vaccine against leishmaniasis, antigens that activate cellular immunity and lead to the production of inter-feron gamma are used (14). Amastigotes proteins have a high ability to induce an immune response, because this phase of the parasite *Leishmania* cause establishment of infection in human body (15).

Our results indicated the existence of both forms of CL in Sistan and Baluchestan Province that may be due to unauthorized entry of foreigners into the country. Leishmaniasis disease is transmitted by sandflies from an infected person, the presence of people who enter the country without any health control is effective on the distribution of the disease, so that in recent years about 19% of the patients in Sistan and Baluchestan were foreigners.

Overall, the large area and the lack of Gene bank from the clinical samples in Sistan and Baluchestan Province were the limitations of our study considered for further studies. On the other hand, we suggest applying transmission electron microscopy (TEM) as a gold standard method to study the molecular microscopy and physiology of the parasite.

## Conclusion

The PCR-RFLP and DNA sequencing methods are valuable techniques for identifying the *Leishmania* species. Higher contamination was related to *L. major* compared with *L. tropica*; these results are consistent with the disease pattern in Sistan and Baluchestan Province, Iran. In order to control the disease, it is suggested to pay more attention to illegal immigration from Pakistan, Afghanistan, and rural areas.
